# Radiation therapy in clinically node positive HER2 positive breast cancer after primary systemic therapy and breast conserving surgery: pooled analysis of TRYPHAENA and NeoSphere trials

**DOI:** 10.1186/s12885-025-14289-4

**Published:** 2025-06-04

**Authors:** Mohamad El-Jammal, Omran Saifi, Jose Bazan, Youssef H. Zeidan

**Affiliations:** 1 Faculty of Medicine, American University of Beirut, Beirut, Lebanon; 2 Department of Radiation Oncology, Mayo Clinic, Jacksonville, FL USA; 3 Department of Radiation Oncology, City of Hope Comprehensive Cancer Center, Duarte, CA USA; 4 Department of Radiation Oncology, Florida International University, Lynn Cancer Institute, Baptist Health South Florida, Boca Raton, FL USA

**Keywords:** Radiation therapy, HER2-positive, Primary systemic therapy (PST), Breast conservation, Pathological complete response (pCR), Locoregional recurrence (LRR)

## Abstract

**Background:**

The benefit of regional nodal irradiation (RNI) following modern primary systemic therapy (PST) in HER2-positive breast cancer (HER2 + BC) remains under investigation. The current study evaluates RNI practice patterns and outcomes based on the pathological response to PST in clinically node-positive (cN+) HER2 + BC.

**Methods:**

TRYPHAENA and NeoSphere are two randomized phase II trials that investigated PST for HER2 + BC. The current study is a pooled analysis of both trials, focusing on cN + patients treated with HER2-targeted PST followed by breast-conserving surgery. The primary goal is to describe patterns of RNI practicein this population and its impact on breast cancer recurrence-free survival (BCRFS) and loco-regional recurrence-free survival (LRRFS).

**Results:**

Our analysis included a total of 90 patients with cN + disease. Complete nodal pathological response was achieved in 53 patients (58.9%). Patients with ypN0 had a 5-year LRRFS of 95.83% whereas patients with ypN + had 5-year LRRFS of 87.43% (*p* = 0.105). RNI was used in 16 ypN0 (48.5%) patients and 17 ypN+ (51.5%) patients. Patients treated with RNI had 5-year LRRFS of 93.4% as compared to 92.5% in the no RNI group (*p* = 0.868). Distant metastasis was detected in 5 patients (5%) with the most common sites being: liver, lung, bone, and CNS. Locoregional recurrence was significantly associated with distant failure (*p* = 0.002).

**Conclusions:**

cN + HER2 + BC patients who achieve ypN0 after PST have excellent locoregional control. In contrast, patients with ypN + tend to have lower locoregional control. The utility of RNI in HER2 + BC warrants further investigation.

## Background

HER2-positive breast cancer (HER2 + BC) is an aggressive subtype of breast cancer characterized by overexpression of the human epidermal growth factor receptor 2 (HER2) protein. Historically, it was associated with poor prognosis [1]. Novel therapies targeting HER2 have shown significant improvements in patients’ outcomes in both early and advanced disease settings [2, 3]. Integration of HER2-directed agents, such as trastuzumab and pertuzumab, with chemotherapy has become the cornerstone of treatment, achieving higher pathological complete response (pCR) rates and better prognoses [4–7]. Such improved oncological outcomes have raised questions regarding the optimal role of radiation therapy (RT), more specifically regional nodal irradiation (RNI), in HER2 + BC, particularly in patients who achieve pCR following primary systemic therapy (PST).

Evidence from historical and recent clinical trials highlight the potential role of RNI in improving locoregional control in patients with clinically node-positive breast cancer (cN+). The pioneering DBCG 82bc trials demonstrated reductions in locoregional recurrence (LRR) and improved survival outcomes with postmastectomy radiotherapy (PMRT) that included RNI [8, 9]. These results withstood the test of time as recently reported in the 30-year update [10]. More specific trials, such as NCIC MA.20 and EORTC 22,922, demonstrated benefits of RNI in reducing LRR and improving survival in cN + patients [11, 12]. The Early Breast Cancer Trialists’ Collaborative Group (EBCTCG) meta-analysis further confirmed these results [13]. However, the advent of modern systemic therapies has renewed questions about the extent and necessity of RNI; signifying a need for more nuanced decision-making especially for patients with low-recurrence-risk profiles. Moreover, current data highlight differences in LRR patterns based on HER2-targeted treatment, as older data did not account for HER2 receptor status or did not include modern anti-HER2 targeted therapies that are standard of practice nowadays [14].

In more recent studies, researchers have evaluated the role of RNI after breast-conserving surgery (BCS) in patients with nodal disease after PST [15]. Preliminary results from the B-51 trial suggest that RNI may be safely omitted in cN1 patients who achieve complete pathological response to PST [16]. Similar findings were recently reported in a prospective registry study (RAPCHEM) and retrospective series [17, 18].

The current study, a pooled analysis of TRYPHAENA and NeoSphere trials, seeks to describe RNI practice patterns and its potential role in cN + HER2 + BC patients treated with modern PST and BCS.

## Methods

### Population characteristics

This pooled secondary analysis utilizes prospectively collected data from two phase II randomized clinical trials—TRYPHAENA and NeoSphere—evaluating PST in HER2 + BC patients. Our cohort was comprised of 90 cN + patients who received anti-HER2 PST, followed by BCS, with a subset of patients receiving RNI.

Access to the raw data was obtained through the Vivli data-sharing platform under Data Request 7583. Detailed description of each trial is available in the original publications [19, 20]. Briefly, TRYPHAENA was designed as a multicenter, open-label, phase II trial that investigated the tolerability and efficacy of trastuzumab and pertuzumab in combination with anthracycline- or carboplatin-based PST regimens in HER2 + BC patients. While NeoSphere was an international, multicenter, open-label phase II study that aimed at evaluating the efficacy and safety of PST regimens including pertuzumab and trastuzumab in a similar patient population. A total of 225 and 392 women were enrolled in the TRYPHAENA and NeoSphere trials, respectively. All included patients were over 18 years old, had operable stage II or higher HER2 + BC, and had tumors larger than 2 cm.

**Fig. 1:**
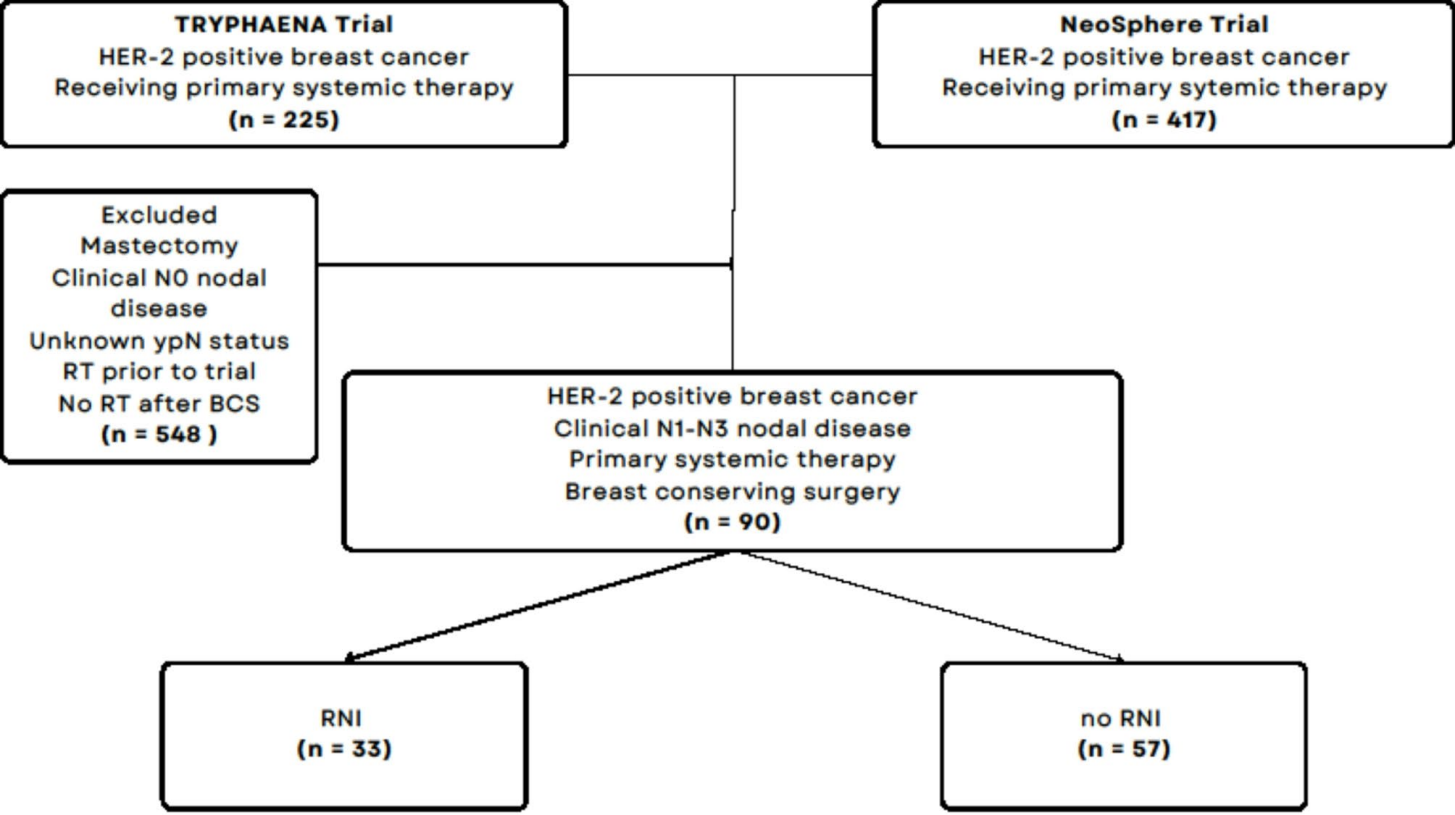
Flow diagram of the study cohort. Abbreviations: HER2 = human epidermal growth factor receptor 2; BCS = breast-conserving surgery; ypN = pathological nodal status at the time of surgery after completion of primary systemic therapy; RNI = regional nodal irradiation

Figure 1 shows the consort diagram of the pooled cohort in the current study. Our eligibility criteria included HER2 + BC patients with evident cN + disease at diagnosis, who received anti-HER2 targeted PST followed by BCS. cN stage was determined by physical exam and/or imaging; nodal biopsies were not mandated. Patients who did not undergo BCS, required mastectomy post BCS, received RT prior to trial start date, did not receive whole breast radiation, or had no cN + disease at diagnosis were excluded. Eligible patients were further categorized according to their pathological status and receipt of RNI.

### Treatment

After completing PST, all patients underwent surgery and continued trastuzumab to complete one year of treatment. Further adjuvant treatment, including RT, chemotherapy, and hormonal therapy, were provided according to local guidelines.

RT, performed between 2007 and 2012, was planned with 3D/CT techniques. RT in both trials was delivered based on physician preference and was not randomized. RNI was defined as radiation to the axillary, supraclavicular, with or without internal mammary lymph nodes. RT targets and doses are summarized in Table 1. A tumor bed boost was delivered to 60 patients with a mean dose of 11 Gy.

**Table 1:** Radiation therapy targets and doses

Radiation Therapy Target	Number of patients	Mean Dose (Gy)
Breast	90	49
Axillary Lymph Nodes	33	47.1
Supraclavicular Lymph Node	33	48.6
Internal Mammary Lymph Nodes	3	43.67
Tumor Bed Boost	60	11
Nodal Boost	6	11

### Outcomes

The primary endpoints of the study were locoregional recurrence-free survival (LRRFS) and breast cancer recurrence-free survival (BCRFS) in patients treated with PST followed by BCS with or without RNI. LRRFS is defined as the duration from the date of enrollment to the occurrence of any local or regional recurrence (LRR), either with or without concurrent distant metastasis (DM). Local recurrence specifically referred to disease recurrence within the ipsilateral breast tissue, while regional recurrence was recurrence in the adjacent lymphatics (specifically: ipsilateral axillary, supraclavicular, infraclavicular, and/or internal mammary lymph nodes). In contrast, BCRFS is defined as the interval from enrollment to the first documentation of any recurrence—whether local, regional, or distant—as well as development of a new ipsilateral or contralateral breast carcinoma, or death from any cause.

### Statistical analysis

STATA statistical software version 17.0 was used to perform all statistical analyses. Data was presented as frequencies and percentages for categorical variables, and as means for continuous variables. We used the chi-square test to compare different demographic, tumor, and treatment-related characteristics between the different groups. Survival outcomes, LRRFS and BCRFS, were estimated by Kaplan-Meier (KM) survival curves, with their differences assessed using the log-rank test. All reported *p*-values are two-sided, with statistically significant difference set at *p* < 0.05.

## Results

Of the combined 642 HER2 + BC patients enrolled in both trials; 90 patients met our inclusion criteria of cN + disease who received PST followed by BCS and RT (Fig. 1). Among those, 33 (36.7%) received whole breast irradiation (WBI) and RNI and 57 (63.3%) received WBI only. The median follow-up period was 5.1 years.

Baseline patient, tumor, and treatment characteristics are presented in Table 2. All patients received HER2-targeted therapy (trastuzumab and/or pertuzumab) and docetaxel with/without other PST followed by BCS. Most patients had negative margins (96.7%) and the majority had an axillary lymph node dissection of at least two levels (88.9%). Patients in the RNI group had more advanced pathological nodal status at surgery (ypN2-3: 27.3% in the RNI group vs. 5.3% in the no-RNI group; *p* = 0.02). However, the remaining clinicopathological and treatment characteristics were balanced between the two groups.

**Table 2:** Baseline characteristics

Characteristics	No RNI	RNI	*p*-value
	*N* = 57	*N* = 33	
	No. (%)	No. (%)	
*Clinical Characteristics*			
**Age**			
< 50	39 (58.2)	21 (63.6)	0.602
≥ 50	28 (41.8)	12 (36.4)	
**Menopause**			
Premenopausal	31 (54.4)	19 (57.6)	0.769
Postmenopausal	26 (45.6)	14 (42.4)	
**ECOG Performance Status**			
0	50 (87.7)	26 (78.8)	0.26
1	7 (12.3)	7 (21.2)	
**Breast Cancer Laterality**			
Left	24 (42.1)	16 (48.5)	0.557
Right	33 (57.9)	17 (51.5)	
**Histology**			
Ductal	55 (96.4)	30 (90.9)	0.5
Lobular	1 (1.8)	1 (3.0)	
Other	1 (1.8)	2 (6.1)	
**Histological Grade**			
Grade 1	1 (1.8)	0 (0.00)	0.775
Grade 2	17 (29.8)	10 (30.3)	
Grade 3	24 (42.1)	12 (36.4)	
Not assessed	15 (26.3)	11 (33.3)	
**Hormone Receptor Status**			
ER (-) and PR (-)	28 (49.1)	20 (60.6)	0.293
ER (+) and/or PR (+)	29 (50.9)	13 (39.4)	
**Tumor Stage**			
T1	0 (0.00)	0 (0.00)	0.353
T2	40 (70.2)	20 (60.6)	
T3	17 (29.8)	13 (39.4)	
T4	0 (0.00)	0 (0.00)	
**Nodal Stage**			
N1	43 (75.4)	23 (69.7)	0.266
N2	13 (22.8)	7 (21.2)	
N3	1 (1.75)	3 (9.1)	
*Treatment Characteristics*			
**Primary systemic therapy**			
Trastuzumab	57 (100)	33 (100)	
Pertuzumab	50 (87.7)	30 (90.9)	0.643
Docetaxel	57 (100)	32 (96.9)	0.186
**Surgical Margins**			
Negative	54 (94.7)	33 (100.0)	0.18
Positive	3 (5.3)	0 (0.00)	
**Axillary Lymph Node Dissection**			
Sentinel lymph node biopsy only	5 (8.8)	1 (3.0)	0.694
Level I only	2 (3.5)	2 (6.1)	
Level I and II	25 (43.9)	16 (48.5)	
Level I, II, and III	25 (43.9)	14 (42.4)	
**Adjuvant Chemotherapy**			
No	9 (15.8)	8 (24.2)	0.488
Yes	43 (75.4)	21 (63.6)	
Not Reported	5 (8.8)	4 (12.1)	
*Pathological Response*			
**Nodal Status at Surgery**			
ypN0	37 (64.9)	16 (48.5)	**0.02**
ypN1	17 (29.8)	8 (24.2)	
ypN2	3 (5.3)	6 (18.2)	
ypN3	0 (0.00)	3 (9.1)	
**Tumor Status at Surgery**			
ypT0	18 (31.6)	13 (39.4)	0.358
ypT1	17 (29.8)	13 (39.4)	
ypT2	9 (15.8)	2 (6.1)	
ypT3	3 (5.3)	0 (0.00)	
ypT4	0 (0.00)	0 (0.00)	
Unknown	10 (17.5)	5 (15.1)	

**Fig. 2:**
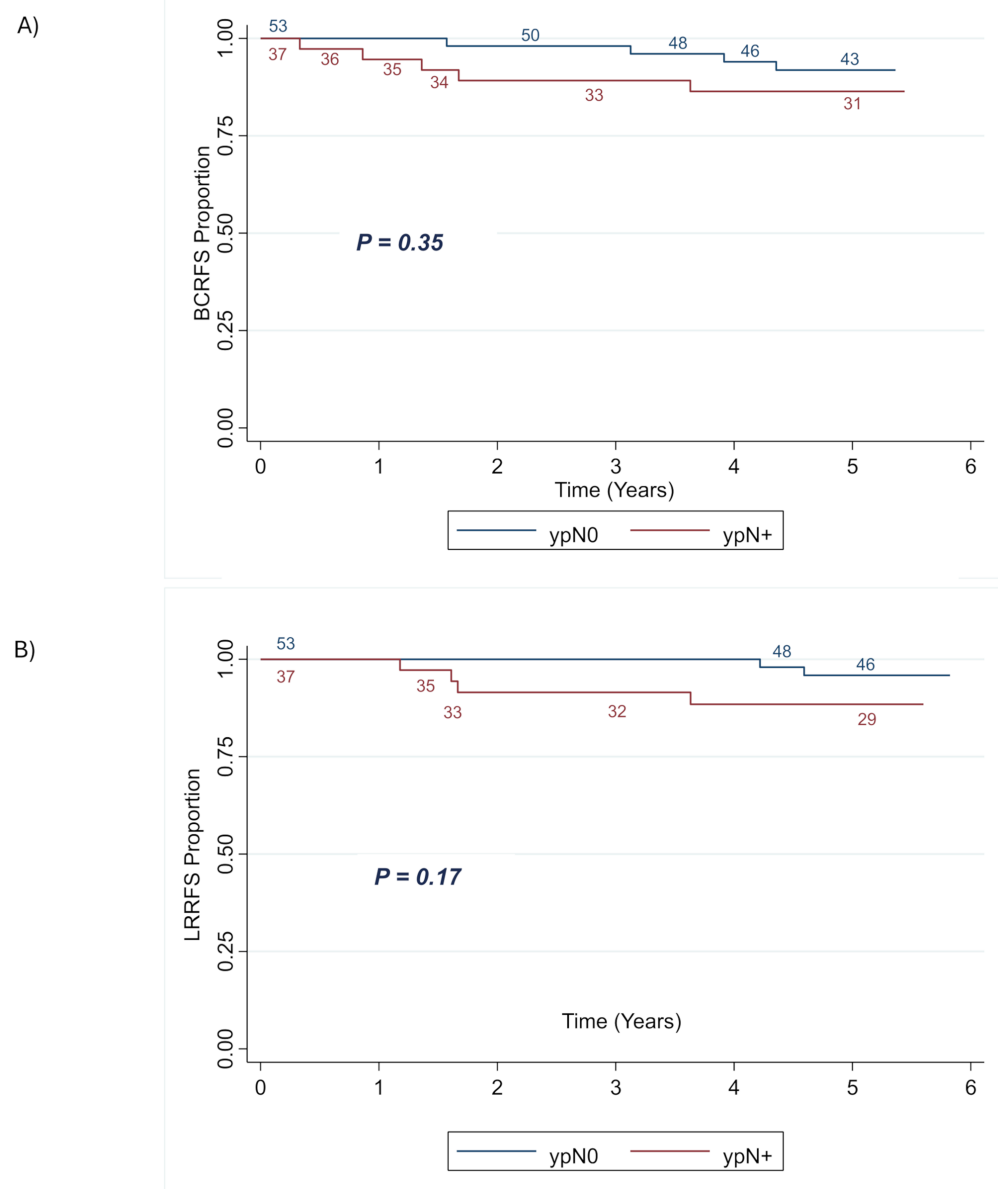
Kaplan-Meier plot showing BCRFS (**A**) and LRRFS (**B**) of patients stratified by nodal pathological response status. Abbreviations: LRRFS = locoregional recurrence-free survival; ypN = pathological nodal status at the time of surgery after completion of primary systemic therapy

At the time of BCS, 53 (58.9%) patients achieved complete nodal pathological response (ypN0), and 37 (41.1%) did not (ypN+). Of those, 25 (27.8%) had ypN1, 9 (10%) ypN2 and 3 (3.3%) ypN3. There were 9 (10%) patients that experienced disease recurrence (loco-regional and/or distant) with 6 having LRR and 5 having DM. The sites of local, regional, and distant recurrences are listed in Table 3. Among the 6 patients with LRR, 2 received RNI and 4 did not. Among the 5 patients with distant recurrence, 4 received RNI and one did not. The 5-year BCRFS was 91.9% in ypN0 and 86.4% in ypN + patients (*p* = 0.35) (Fig. 2A). The 5-year LRRFS was 95.9% in ypN0 and 88.5% in ypN+ (*p* = 0.17) (Fig. 2B).

**Table 3:** Locoregional and distant recurrences

**Number of patients with locoregional recurrence**				**Number of patients with distant recurrence**	
6				5	
**Number of locoregional recurrence events**				**Number of distant recurrence events**	
6				6	
**Site of locoregional recurrence**				**Site of distant recurrence**	
Ipsilateral breast tissue	5 (83%)			CNS	1 (17%)
Ipsilateral breast skin	1 (17%)			Liver	2 (33%)
				Bone	2 (33%)
				Lung	1 (17%)

**Fig. 3:**
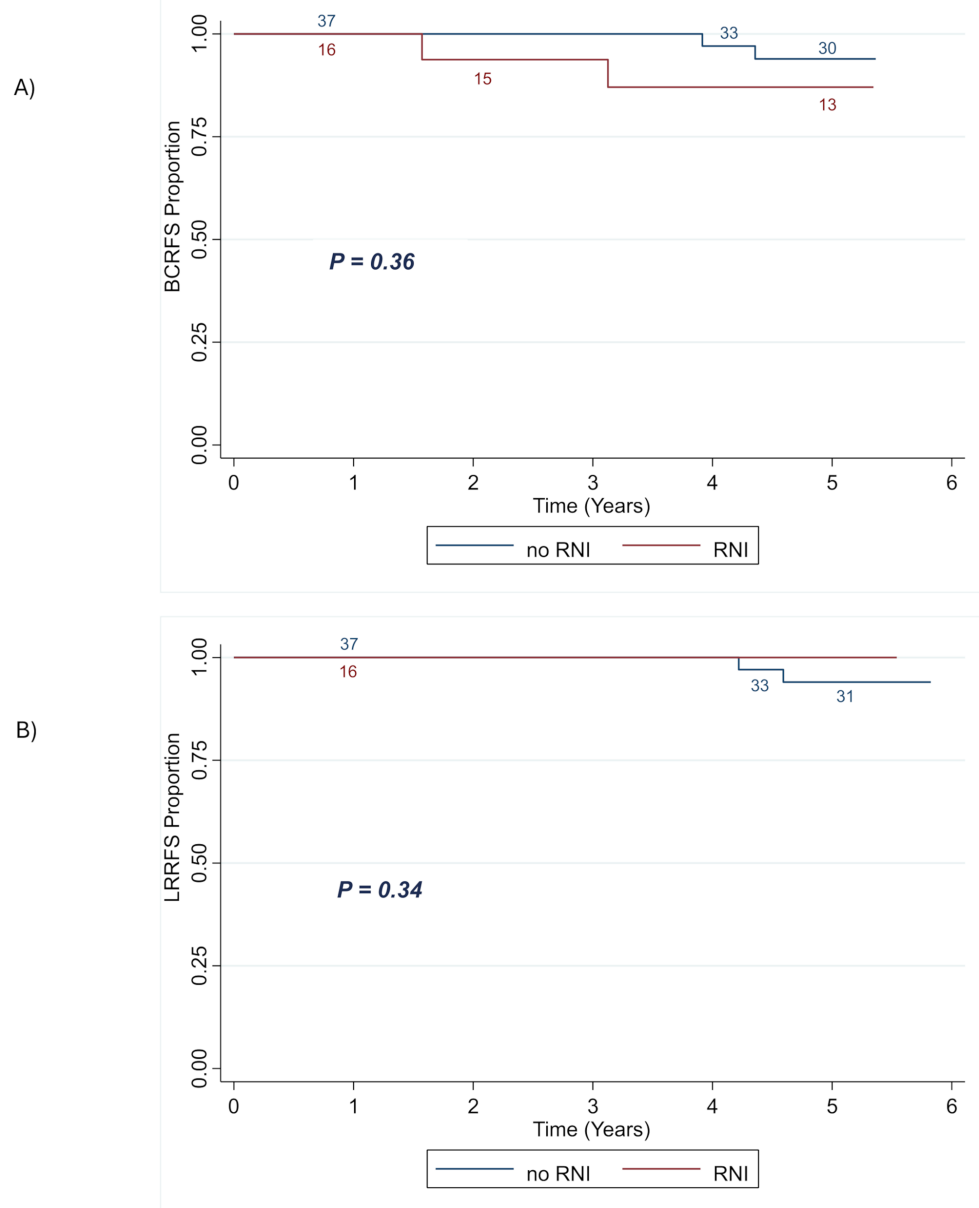
Kaplan-Meier plot showing BCRFS (**A**) and LRRFS (**B**) of patients with ypN0 stratified by the receipt of RNI. Abbreviations: BCRFS = breast cancer recurrence-free survival; LRRFS = locoregional recurrence-free survival; RNI = regional nodal irradiation

**Fig. 4:**
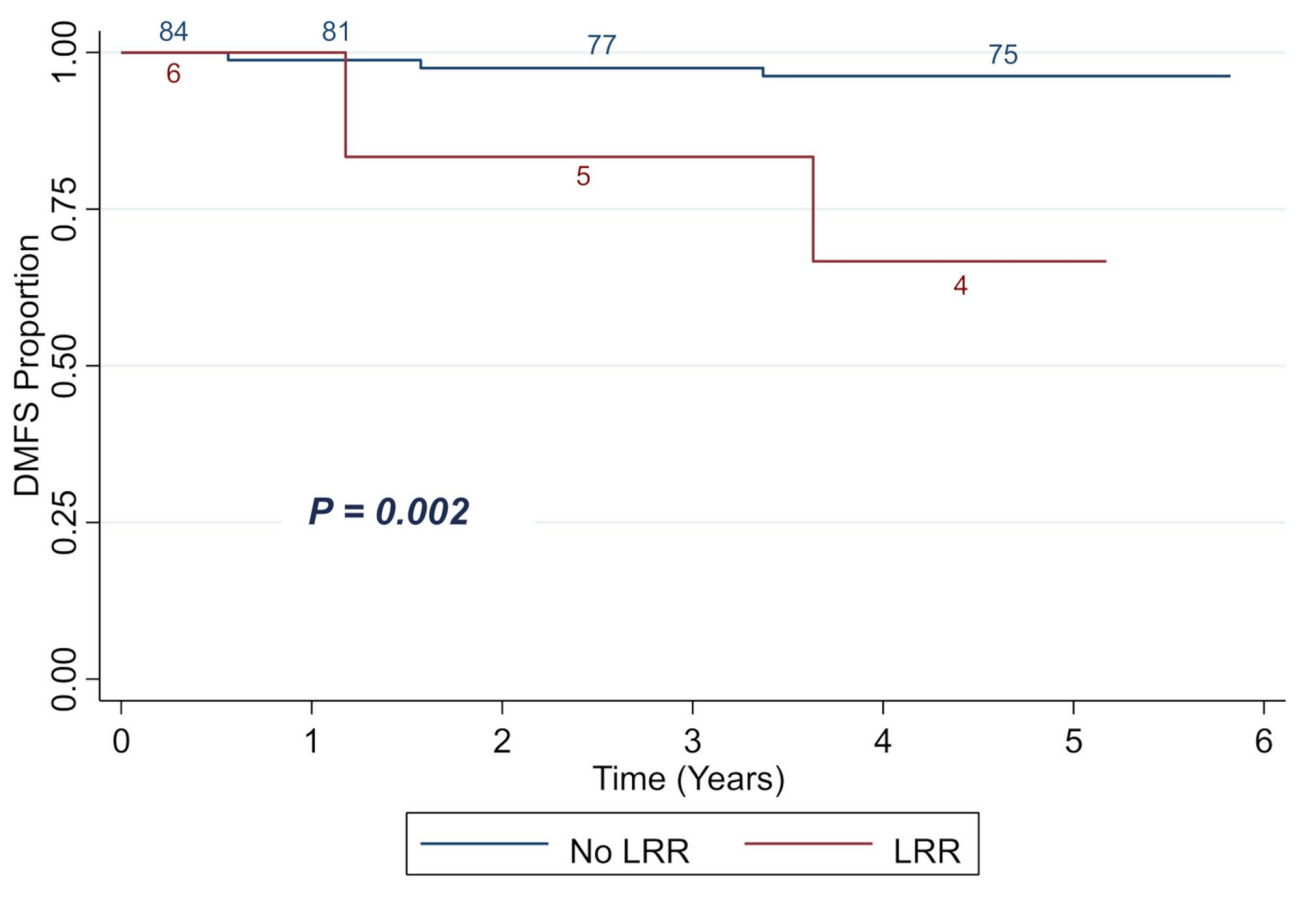
Kaplan-Meier plot showing DMFS proportion of all patients stratified by LRR status. Abbreviations: DMFS = distant metastasis-free survival; LRR = locoregional recurrence

Among the 53 patients with ypN0 at the time of BCS, 16 (30.2%) received RNI and 37 (69.8%) did not. Adjuvant chemotherapy was given to 6 ypN0 patients (37.5%) in the RNI group and to 26 patients (70.3%) of the no RNI group. There were 2 patients (12.5%) in the RNI group and 2 patients (5.4%) in the no-RNI groups that experienced breast cancer recurrence. The 5-year BCRFS was 87.1% in the RNI group and 93.9% in the no-RNI group (*p* = 0.36) (Fig. 3A). There were no patients in the RNI group that experienced LRR, while two patients in the no-RNI group did. This translated to a 5-year LRRFS of 100% in the RNI group and 94.03% in the no-RNI group (*p* = 0.34) (Fig. 3B). Importantly, patients with no LRR, had a 5-year distant-metastasis free survival (DMFS) of 96.2%, as compared to 66.7% in patients with confirmed LRR (*p* = 0.002) (Fig. 4). Univariate analysis of multiple clinical factors, including RNI, did not show significant correlation with LRR or BCR (Table 4).

**Table 4:** Univariate Cox proportional hazard regression model of LRR and BCR

Variable		LRR		BCR	
		HR (95% CI)	*p* value	HR (95% CI)	*p* value
**Age**	< 50	Reference		Reference	
	> 50	1.24 (0.28–5.53)	0.78	1.74 (0.35–8.64)	0.5
**Menopausal Status**	Premenopausal	Reference		Reference	
	Postmenopausal	0.94 (0.21–4.21)	0.94	1.62 (0.43–6.03)	0.47
**ECOG**	0	Reference		Reference	
	1	4.85E-16	0.13	0.65 (0.08–5.17)	0.66
**Laterality**	Left	Reference		Reference	
	Right	2.25 (0.44–11.59)	0.33	2.93 (0.61–14.11)	0.14
**Histology**	Ductal Carcinoma	Reference		Reference	
	Lobular Carcinoma	2.07 (0.69–6.31)	0.27	1.76 (0.59–5.28)	0.37
**Hormone Receptor Status**	ER(-) and PR(-)	Reference		Reference	
	ER(+) and/or PR(+)	0.81 (0.18–3.61)	0.78	0.52 (0.13–2.10)	0.35
**Clinical T Stage**	T2	Reference		Reference	
	T3	0.40 (0.04–3.10)	0.36	0.55 (0.12–2.67)	0.44
**Clinical Nodal Stage**	N1	Reference		Reference	
	N2	0.63 (0.07–5.42)		0.91 (0.19–4.37)	
	N3	2.38 (0.28–20.37)	0.66	1.55E-15	**0.63**
**RNI**	No	Reference		Reference	
	Yes	0.87 (0.15–4.73)	0.87	2.25 (0.61–8.40)	0.22
**Axillary Lymph Node Dissection Levels**	Sentinel biopsy	Reference		Reference	
	I only	1.13E-07		2.26E-06	
	I-II	5.17E + 08		4.17E + 08	
	I-II-II	7.01E + 08	0.37	8.83E + 08	0.175
**Nodal pCR**	Yes	Reference		Reference	
	No	1.74 (0.80–3.77)	0.19	1.84 (0.49–6.86)	**0.36**
**Tumor pCR**	Yes	Reference		Reference	
	No	2.12 (0.46–9.68)	0.32	2.44 (0.65–9.19)	0.18

## Discussion

RT after breast cancer PST remains under investigation. The current study is one of the largest to evaluate RT patterns of practice and outcomes in cN + HER2 + BC after BCS. Our analysis included 90 patients, treated with whole breast irradiation after BCS with one third receiving additional RNI. Patients with ypN0 had improved rates of LRRFS as compared to ypN+ (95.8% vs. 87.4%). Addition of RNI had minimal impact on LRRFS and BCRFS in patients with ypN0 following PST.

Regional nodal irradiation is widely utilized in cN + breast cancer patients undergoing upfront surgery, however its utility after primary systemic therapy remains controversial. The MA.20 and EORTC 22,922 trials demonstrated that RNI can reduce locoregional and distant recurrences [11, 12]. This impact of RNI on oncological outcomes was recently confirmed by a meta-analysis from the EBCTCG [13]. However, secondary analysis of the ALTTO trial failed to demonstrate a benefit for RNI in HER2 + BC patients [21]. The authors attribute these findings to the use of modern HER2 targeted systemic therapy in the adjuvant setting. The wide adoption of neoadjuvant therapies coupled to the improved pCR rates in HER2 + BC warrant further investigation into the role of RNI. On the other hand, RNI is associated with increased toxicity risks such as lymphedema and pneumonitis [12]. Therefore, the decision to deliver RNI needs to carefully balance individual patient’s benefits and risks.

When compared to ypN0 patients, our results indicate that patients with residual nodal disease have lower LRRFS (87.4% vs. 95.8%) and BCRFS (86.4% vs.91.9%). This finding is supported by several studies suggesting increased risk of recurrence in patients with residual nodal disease after PST [18, 22, 23]. Addition of RNI in ypN + patients was recently shown to improve LRR [18, 24] and overall survival [24, 25]. A risk adapted approach, whereby RNI was tailored according to level of residual axillary disease, was recently proven in the RAPCHEM trial to have low 5-year LRR (< 4%) [17]. It is worth noting that the majority of the patients (88.9%) in the current study underwent axillary dissection (ALND). However, recent studies suggest a low incidence of axillary recurrence (0.38–1%) in patients with limited residual axillary disease treated with sentinel lymph node (SLN)/ targeted axillary dissection (TAD) and axillary radiation [26]. Omission of ALND in this setting is currently being investigated in the Alliance 011202, OPBC-03/TAXIS, AXSANA, ADARNAT and NEOSENTITURK trials [26–30].

Patients with complete nodal pathological response had excellent LRRFS and BCRFS outcomes in our analysis. No significant benefit for RNI was noted in this subgroup, supporting treatment de-escalation in this patient population. Two recent retrospective studies reached a similar conclusion for HER2 patients receiving modern systemic therapies [15, 18]. Analysis from NSABP studies also reported lack of association between RNI and improved oncological outcomes in ypN0 patients [25]. However, a pooled analysis from German trials including all subtypes demonstrated locoregional control benefit for RNI in ypN0 subgroup [31]. Definitive answer to this question and others will be provided by the NSABP-B-51 trial, which has been presented in abstract format only [16]. Of note 56.5% of enrolled patients were HER2 positive. Other trials with a similar design, such as ATNEC, are also underway [32].

Despite the current work presenting real world data on HER2 + BC patients treated with PST, this analysis has several limitations. RNI was not standardized thus risking selection bias. Second, the study may not be powered enough to detect a small benefit for RNI due to the small sample size and low number of events. For instance, the EBCTCG analysis of RNI reported a 2.4% gain in breast cancer recurrence at 15 years in a meta-analysis of more than 14 thousand patients [13]. Another limitation is our small sample size. Other datasets, besides NeoSphere and TRYPHAENA, are needed to validate our findings. Moreover, due to low number of events, no subgroup analysis was done according to ypN + subcategories. Finally, the study did not include newer HER2 targeting therapies such as T-DM1 which have proven efficacy in HER2 patients with residual disease [33].

In conclusion, our findings show patterns of RT practice of two large trials with no evidence of significant benefit from RNI for ypN0 patients. These results, while not definitive, contribute to the evolving body of evidence supporting a more tailored approach to RNI, incorporating pathological response and molecular subtypes. Moreover, given the limitations inherent to the available data, these findings should be interpreted as hypothesis-generating. Future and ongoing trials will be critical for shaping the role of RT as well as axillary management in the setting of PST. Clinicians must weigh the potential benefits of RNI against the risks of overtreatment to provide personalized, evidence-based care.

## Data Availability

The data presented in this paper was acquired from raw data in clinical trials. The raw data from TRYPHAENA and NeoSphere are not publicly available to preserve individual privacy and protect patient confidentiality. Data is locked by an independent, non-profit organization that has developed a global data-sharing and analytics platform (Vivli).

## Abbreviations

ALND: Axillary Lymph Node Dissection
BCRFS: Breast Cancer Recurrence-free Survival
BCS: Breast-conserving Surgery
cN: Clinical Nodal Status
DM: Distant Metastasis
DMFS: Distant Metastasis-free Survival
HER2 + BC: HER2-positive Breast Cancer
HER2: Human Epidermal Growth Factor Receptor 2
LRR: Locoregional Recurrence
LRRFS: Locoregional Recurrence-free Survival
pCR: Pathological Complete Response
PMRT: Post-mastectomy Radiation Therapy
PST: Primary Systemic Therapy
RNI: Regional Nodal Irradiation
RT: Radiation Therapy / Radiotherapy
SLN: Sentinel Lymph Node
TAD: Targeted Axillary Dissection
WBI: Whole Breast Irradiation
ypN: Pathological Nodal Status at Surgery
